# Effect of electromagnetic field radiation on transcriptomic profile and DNA methylation level in pig conceptuses during the peri-implantation period

**DOI:** 10.1038/s41598-025-98918-9

**Published:** 2025-04-23

**Authors:** Anita Franczak, Agata Zmijewska, Ewa Monika Drzewiecka, Wiktoria Kozlowska, Pawel Wydorski, Lukasz Paukszto, Tiziana L. Brevini

**Affiliations:** 1 Department of Animal Anatomy and Physiology, Faculty of Biology and Biotechnology, University of Warmia and Mazury in Olsztyn, Oczapowskiego Str. 1A, 10-719 Olsztyn, Poland; 2 Department of Gamete and Embryo Biology, Institute of Animal Reproduction and Food Research, Polish Academy of Sciences in Olsztyn, Tuwima 10, 10-748 Olsztyn, Poland; 3 Department of Botany and Nature Protection, Faculty of Biology and Biotechnology, University of Warmia and Mazury in Olsztyn, Plac Łódzki 1, 10-719 Olsztyn, Poland; 4 Laboratory of Biomedical Embryology, Department of Veterinary Medicine and Animal Sciences, Center for Stem Cell Research, Università degli Studi di Milano, 20134 Milan, Italy

**Keywords:** Extremely low-frequency electromagnetic field, Next-generation sequencing, DNA methylation, Conceptuses, Pig, Molecular biology, Physiology, Reproductive biology

## Abstract

Extremely low-frequency electromagnetic field (ELF-EMF) radiation alters the steroidogenic activity of porcine conceptuses during the peri-implantation period. This study investigated whether short exposure (2 h) to ELF-EMF radiation may induce changes in the transcriptomic profile of conceptuses and their DNA methylation levels, with a focus on the promoter regions of differentially expressed genes of interest. Porcine conceptuses were collected on days 15–16 of pregnancy, preincubated, and then exposed in vitro to ELF-EMF at a frequency of 50 Hz. ELF-EMF treatment affected the expression of 21 protein-coding transcripts, including *solute carrier family 34 member 1* (*SLC34A1*), *hydroxysteroid 17-beta dehydrogenase 2* (*HSD17B2*), *apolipoprotein M* (*APOM*), *regucalcin* (*RGN*), *heat shock protein family A (Hsp70) member 6* (*HSPA6*), *fibrinogen beta chain*, and *fibrinogen gamma chain* (*FGG*). There were evaluated 21 GO annotations for biological process terms, nine GO annotations for cellular component terms and two KEGG pathways. The ELF-EMF exposure increased approximately 16 times genomic DNA methylation in conceptuses. ELF-EMF-induced changes in methylation were observed in the promoter regions of *APOM, HSD17B2, FGG,* and *SLC34A1*. The results determined 116 predicted single nucleotide variant substitutions within RNA editing sites. In conclusion, ELF-EMF radiation appears to interact with DNA methylation levels and to affect the expression of genes involved mainly in cellular homeostasis, conceptuses development, and attachment, suggesting the need for further investigation to elucidate better the impact of ELF-EMF exposure on the physiology of early conceptuses.

**Supplementary Information:**

The online version contains supplementary material available at 10.1038/s41598-025-98918-9.

## Introduction

The extremely low frequency electromagnetic field (ELF-EMF), ranging from 1 to 300 Hz is considered as a hazardous and stress factor in everyday human and animal environment^1–3^. Most of the population is chronically exposed to the ELF-EMF at the frequency of 50 Hz or 60 Hz and magnetic flux densities of less than 1 µT, although humans living or working near high-current machines may be exposed at levels exceeding much more over 1 mT magnetic flux density. For example, magnetic flux density may range from 1 to 6 mT for induction heating, 1 to 10 mT for electrolytic processes, and from 1 to 16 mT for therapeutic equipment^4^ which are used in modern industry and medicine, respectively. Moreover, in the therapy, there are also used devices emitting fields with magnetic induction up to 30 mT^4^. Interestingly, there are also much stronger sources of the ELF-EMF radiation, generated electromagnetic induction in Tesla. For example, multipurpose electromagnetic stimulation devices, which are used in modern medicine to relieve pain diseases, or for neural and/or muscle control, generate magnetic induction around 7 T^4,5^. Therefore, it is worth noting that people and animals are surrounded by the ELF-EMF at various levels of frequency and magnetic induction. A short treatment duration (2 h) of ELF-EMF exposure at the frequency of 50 Hz is the most common and falls within the permissible electromagnetic field range in the environment^1,2,4^. The International Agency for Research on Cancer has classified the ELF-EMF as leading to health risks and possible carcinogen^6^.

The peri-implantation period is critical for pregnancy success, with up to 30% embryo loss possible but the reasons are not fully understood^7,8^. It is disturbing that many women may unknowingly undergo physical therapies involving electromagnetic fields during the peri-implantation period, which could increase the likelihood of pregnancy loss^3^. Therefore, understanding the ELF-EMF-induced molecular effects is crucial for developing risk mitigation guidelines. Moreover, knowing the consequences of the ELF-EMF radiation is needed to develop future strategies to optimize breeding environments and improve reproductive outcomes in animal farming, including pig breeding.

It was documented that environmental exposure to the ELF-EMF may express adverse effects on female reproduction. Observed consequences depend on the ELF-EMF intensity, frequency and duration of treatment^5,9–13^. The results of studies in animal models revealed that exposure to the ELF-EMF may be detrimental to uterine and conceptuses activity affecting steroid hormone synthesis and secretion^11,14–17^. Moreover, the ELF-EMF was found to affect proliferation^18–20^, differentiation^21,22^, migration^18^ and apoptosis^23^ in different cell types. Interestingly, it was also documented that ELF-EMF radiation may evoke epigenetic changes, including DNA methylation, histone modifications, and microRNA biogenesis^24–26^. In particular, in the consequence of ELF-EMF radiation transcriptomic activity in the endometrium was mainly down-regulated whereas up-regulated in the myometrium^9,10^.

Considering potentially high risks associated with the consequences of ELF-EMF exposure on female reproductive tissues there is an urgent need to understand the impact of the ELF-EMF exposure on conceptuses. The current study breaks new ground by examining the ELF-EMF impact on transcriptomic profile and DNA global methylation and selected promotor regions methylation changes in conceptuses. It is important to highlight that while previous research focused on the consequences of ELF-EMF have been conducted mainly using small laboratory animals^27^, the current study focuses on conceptuses of pigs during the critical peri-implantation period. Obtained results fill a significant gap in our understanding of the ELF-EMF affects conceptuses transcriptome and epigenetics regulations and provider significance in the broader context of reproductive health.

Due to physiological and anatomical similarities between pigs and humans, including those related to reproduction i.e. fertilization, early embryonic development, pregnancy and reproductive diseases, pig conceptuses constitute an excellent model for understanding molecular aspects of human reproduction^28,29^. The prolonged apposition and attachment phase (without invasion into the luminal epithelium) makes the pig an appropriate candidate model for studying the early phases of implantation at the molecular level^30^. However, particularly implantation timing and mechanisms constitute also some differences as human embryos implant readily upon hatching^28^. Despite differences in the type of implantation between the pigs and humans, pigs are still very good candidate model to study early phases of implantation^28,30^ and an outstanding animal model for biomedical research in humans^29,31^. To obtain a comprehensive view of the impact of the ELF-EMF on peri-implantation conceptuses, transcriptome profiling, RNA editing site prediction and alternative splicing (AS) events, genomic DNA methylation, and the amplification of methylated and unmethylated promoters of selected differentially expressed genes were determined in porcine conceptuses after their exposure to the ELF-EMF at the frequency of 50 Hz in vitro. The obtained results documented the potential of the EMF-ELF to affect the transcriptomic profile and epigenetic regulation in conceptuses during the peri-implantation period and provide a better understanding of ELF-EMF exposure-related consequences.

## Results

### The statistics of RNA sequencing

**Fig. 1:**
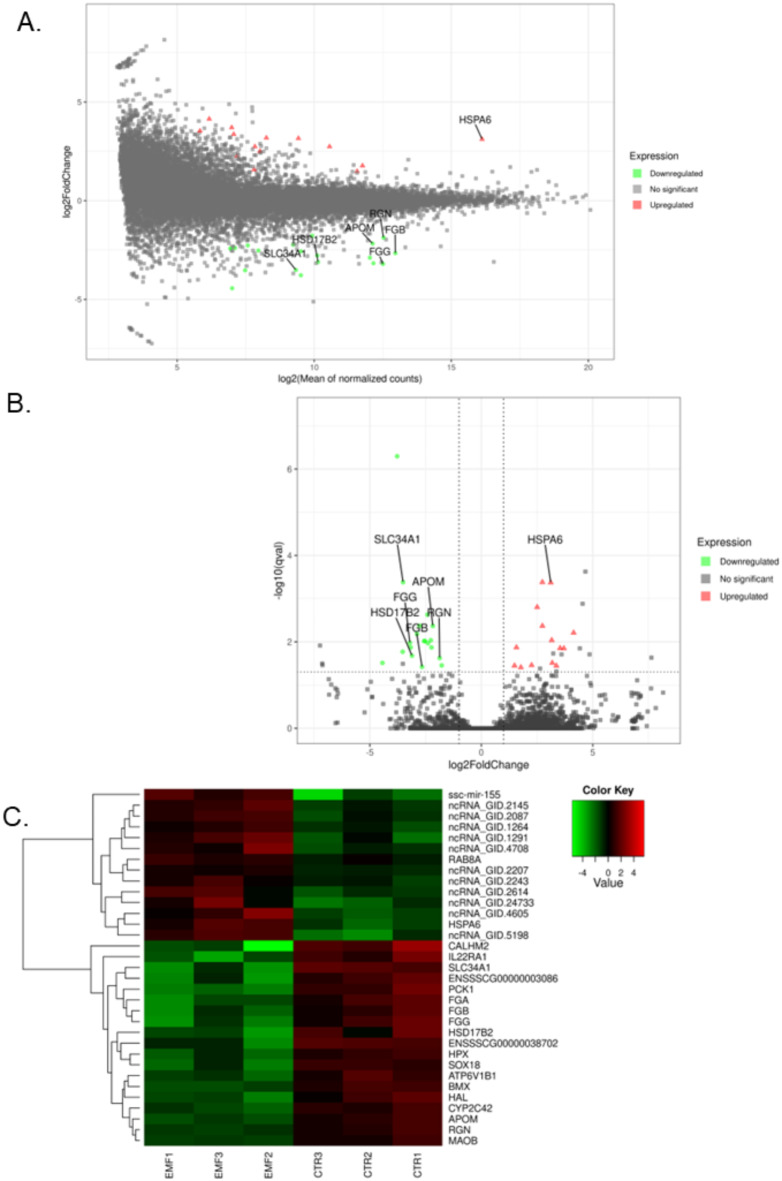
The MA plot, volcano plot and heatmap of expression data. (**A**) The MA plot shows the log scale of the fold changes (log2FCs) on the Y axis and the mean expression rate on the X axis. (**B**) The volcano plot describes the logFC on the X-axis and the negative logarithmic adjustment of the p value on the Y-axis. The red triangles represent upregulated TARs, and the green dots represent downregulated TARs. (**C**) Heatmap of expression data. The columns represent individual libraries; the rows indicate the gene symbols of the DEGs. The color key scale was applied for visualization of the expression values (FPKM) of each biological replicate.

The overall statistics of the RNAseq data were constructed for six cDNA libraries (three extracted from conceptuses treated with the ELF-EMF and three extracted from the controls). After sequencing, 171,243,550 raw paired reads were obtained, with an average of 28.5 mln per sample. The 152,900,921 filtered reads were mapped to the pig genome (version Ss11.1.98), and the unique mapping rate ranged between 83.1% and 93.1%. The distribution of mapped reads to gene structures was as follows: 53.49% of the read pairs mapped to coding sequences, 6.45% mapped to introns, 22.57% aligned to untranslated regions, and the remaining 17.49% mapped to intergenic regions. An overview of the changes in gene expression is illustrated in MA, volcano plots, and heatmaps (Fig. 1A–C).

### Identifying differentially expressed genes (DEGs)

A 2 h treatment duration with the ELF-EMF at 50 Hz affected the expression of 33 differentially expressed transcriptionally active regions** (**DE-TARs), among which 21 were protein-coding genes. The other 12 were represented by nonclassified differentially expressed non-coding RNA (DE-ncRNAs). Among the 19 downregulated TARs, 17 were assigned gene names, whereas among the upregulated TARs, protein-coding annotation has 2 out of 14 DE-TARs candidates. The DEGs whose expression was upregulated in response to the ELF-EMF treatment were heat shock protein family A (Hsp70) member 6 (*HSPA6*) and member RAS oncogene family (*RAB8A*), whereas top five genes whose expression was downregulated with the highest log2FC values were calcium homeostasis modulator family member 2 (*CALHM2*), phosphoenolpyruvate carboxykinase 1 *(PCK1*), interleukin 22 receptor alpha 1 (*IL22RA1*), solute carrier family 34 member 1 (*SLC34A1*) and the fibrinogen gamma chain (*FGG*). A list of all the evaluated DEGs in porcine conceptuses exposed to the ELF-EMF at 50 Hz for 2 h can be found in Supplementary Table S1.

### Functional ontology annotations

**Fig. 2:**
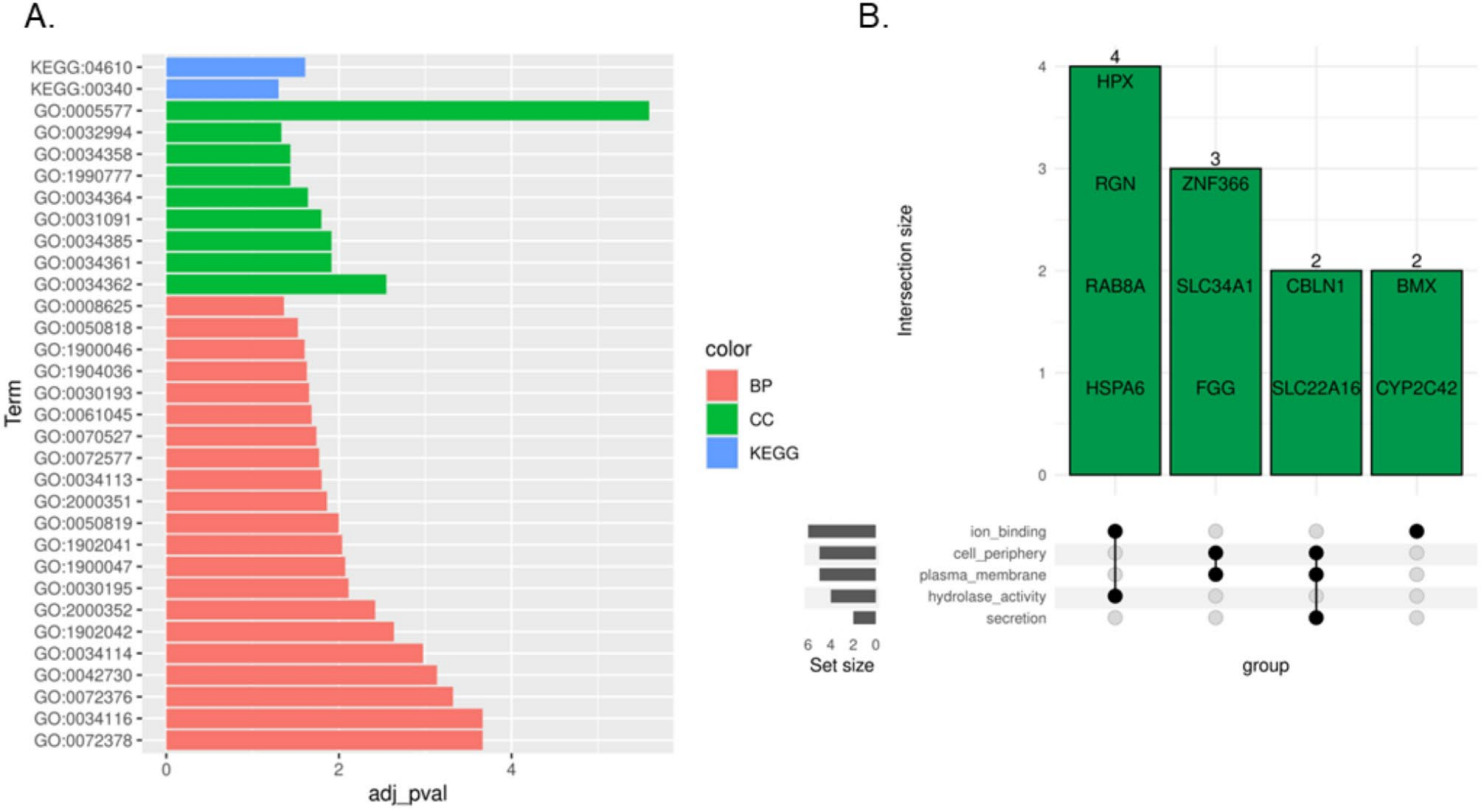
Gene Ontology and Kyoto Encyclopedia of Genes and Genomes (KEGG) pathway analyses. (**A**) Bar chart of the ontology terms (biological process; BP and cellular components, CC and signaling pathways) detected during Gene Ontology and Kyoto Encyclopedia of Genes and Genomes (KEGG) pathway analyses associated with differentially expressed genes (DEGs) that were altered in the ELF-EMF-treated porcine conceptuses. The scale bars refer to the logarithmic scale of the adjusted p value in the enrichment analysis. The color links merge genes with GO and KEGG annotations on the right side (**A**). (**B**) The gene set enrichment analysis (GSEA) results are plotted as an UpSet diagram describing the intersection of the five ontology terms. The black dots joined by lines represent GO terms with common DEG assignments (green bars). The horizontal grey bars indicate the number of DEGs assigned to a particular GO (**B**).

The DEGs were assigned to the functional ontology annotations and there were evaluated 21 GO annotations for biological process (BP) terms, including blood coagulation, fibrin clot formation, positive regulation of heterotypic cell‒cell adhesion, protein activation cascade and fibrinolysis, and nine GO annotations for cellular component (CC) terms, including fibrinogen complex*,* low-density lipoprotein particle, high-density lipoprotein particle, very-low-density lipoprotein particle, triglyceride-rich plasma lipoprotein particle, platelet alpha granule and high-density lipoprotein particle. Two KEGG pathways, i.e., complement and coagulation cascades (KEGG:04610) and histidine metabolism (KEGG:00340), nine REACTOME pathways and four HP pathways (Supplementary Table 2A), were also identified. The evaluated GO and KEGG pathways are presented in Fig. 2A.

GSEA (Fig. 2B, Supplementary Table 2B) revealed five significant functional ontologies, i.e., hydrolase activity, plasma membrane, cell periphery, secretion, and ion binding, represented by the following DEGs: *HSPA6, RAB8A,* regucalcin (*RGN*)*,* hemopexin *(HPX), FGG, SLC34A1,* zinc finger protein 366 *(ZNF366),* solute carrier family 22 member 16 *(SLC22A16),* cerebellin 1 precursor *(CBLN1),* cytochrome P450 C42 *(CYP2C42)* and BMX non-receptor tyrosine kinase (*BMX)*.

### RNA editing site prediction

**Fig. 3:**
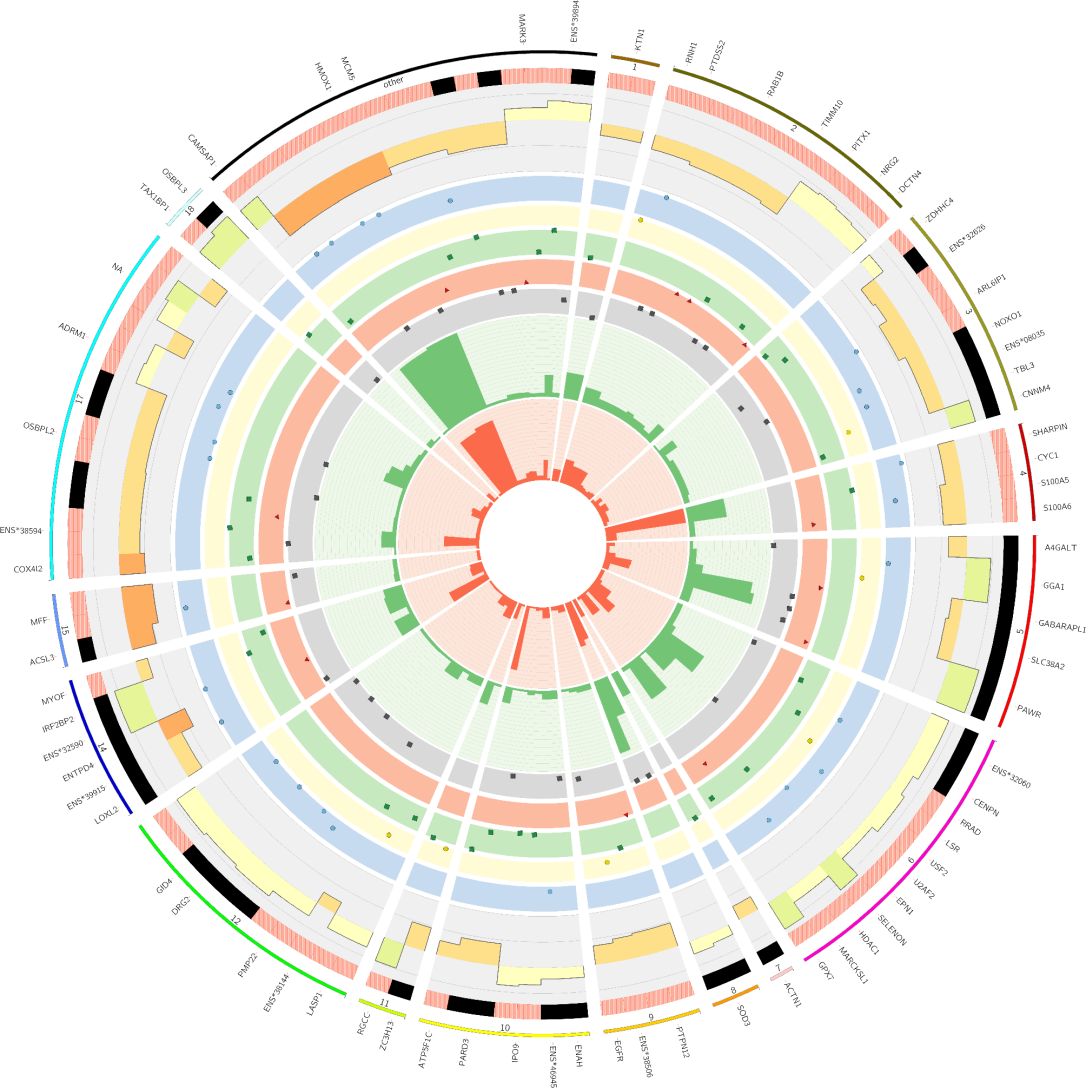
RNA editing candidates exposed to EMF in porcine embryos. The external circle represents the porcine chromosomes (without chr13 and 16—no DE editing sites) and other unclassified scaffolds (other), where the region length is proportional to the number of editing substitutions. The second track (black and red lined blocks) presents an abundance of adenine to inosine and cytosine to thymine (A-to-I and C-to-T) types of RNA editing sites. The third track, the histogram, depicts the difference in the alternative allele fraction (ΔAAF) between the EMF-treated samples and the controls. The next five middle scatter plots present the localization of RNA editing substitutions upstream and downstream (blue dots), missense (yellow dots), synonymous (green square), and intron (red triangle) UTRs (grey square); the vertical axis on each scatter plot shows the normalized FDR. The two inner tracks show the sums of coverage for the alternative (red histogram) and reference variants (green histogram) in all the RNA-seq libraries. Figure created with Circos software.

In the conceptuses tissue exposed in vitro to 50 Hz of the ELF-EMF, the variant calling analysis revealed 159,014 SNVs. After GATK filtration, for downstream analysis, the 33,895 SNVs were eliminated, and subsequently, following porcine genome screening, 111,148 substitutions positioned near bidirectional genes, simple sequence repeats, paralogs, and those in the vicinity of splice junction regions were removed/filtered out. The 13,971 SNVs with alternate allele frequencies (AAFs) were obtained in a minimum of half of the RNA-seq libraries. Next, 3,029 substitutions annotated as SNPs and possessing too high levels of alternate alleles frequency (AAF > 0.7) in any experimental sample were removed. Next, because of the filtration procedure, 170 substitutions were found, which indicated a significant (FDR < 0.001, *∆*AAF > 0.1 and < -0.1) imbalance in allele expression between the ELF-EMF-treated and control conceptuses samples. Finally, according to the variant effect predictor (VEP) annotation, 116 candidates in the conceptus transcriptome were assigned as canonical RNA substitutions (A to I and C to T) (Fig. 3; Supplementary Table S3). The VEP assigned RNA editing candidates to the following main variation consequences: 11—upstream gene, 3—5′UTR, 30—3′UTR, 6—missense, 31—synonymous, 10—intron variant calling, and 17—downstream variants. Among these 17 downstream variants, 3 out of the identified RNA editing variants were found within the downstream gene variant of heme oxygenase 1 *(HMOX1)*. The SNVs were assigned to two enriched CCs: the cytoplasm and the organelle membrane. KEGG analysis revealed two processes, Huntington’s disease and Amyotrophic lateral sclerosis, whereas the REACTOM database revealed one item: EGFR downregulation, with three genes, protein-tyrosine phosphatase (PTP)-PEST *(PTPN12)*, epidermal growth factor receptor *(EGFR),* and epsin 1 *(EPN1)* (Supplementary Table S4).

### Differential alternative splicing (AS) events

**Fig. 4:**
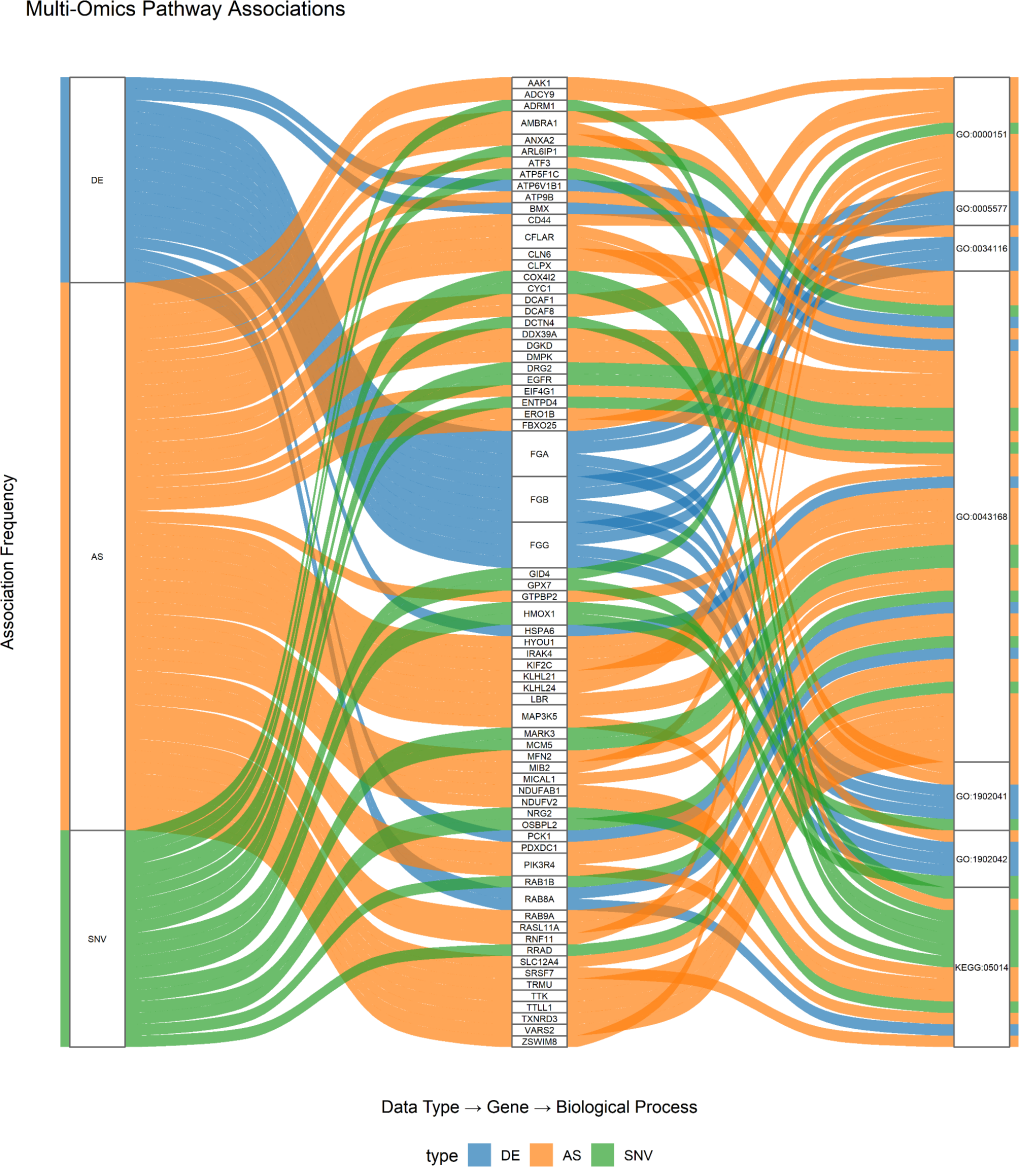
The Sankey diagram illustrates the relationships between genes identified in differential expression (DE), alternative splicing (AS) and single nucleotide variant (SNV) analyses, and their enriched Gene Ontology (GO) terms and KEGG pathway. The left side of the diagram constitutes DE, AS, and SNV layer; these are linked to specific genes, and the relationship between the genes and data type. Finally, on the right, the GO terms and KEGG pathway associated with the genes are presented. This shows the frequency of association between each data type, individual genes, and these functional terms.

During the analysis, using approach rMATs we found 77,323 splicing events (Supplementary Table S5), whereas the comparison of the samples obtained from the control and the ELF-EMF-treated conceptuses resulted in 348 differentially alternative splicing (DAS) events. The AS method classified the 177 DAS events as skipping exons (SE), 107 DAS events as retained introns (RI), 27 DAS events as alternative 5′ splice sites (A5SS), 22 DAS events as alternative 3′ splice sites (A3SS) and 15 DAS events as mutually exclusive exons (MXE). All the identified DAS events were assigned to 179 protein-coding genes. Sankey plot summarized the results from DEGs, SNVs and AS analyses and anion binding (GO:0043168), fibrinogen complex (GO:0005577), positive regulation of heterotypic cell–cell adhesion (GO:0034116), ubiquitin ligase complex (GO:0000151), regulation of extrinsic apoptotic signaling pathway via death domain receptors (GO:1902041), amyotrophic lateral sclerosis (KEGG:05014) were enriched. The visualization of the Sankey diagram is presented in Fig. 4 and Supplementary Table S6.

### VENN analysis results

**Fig.5:**
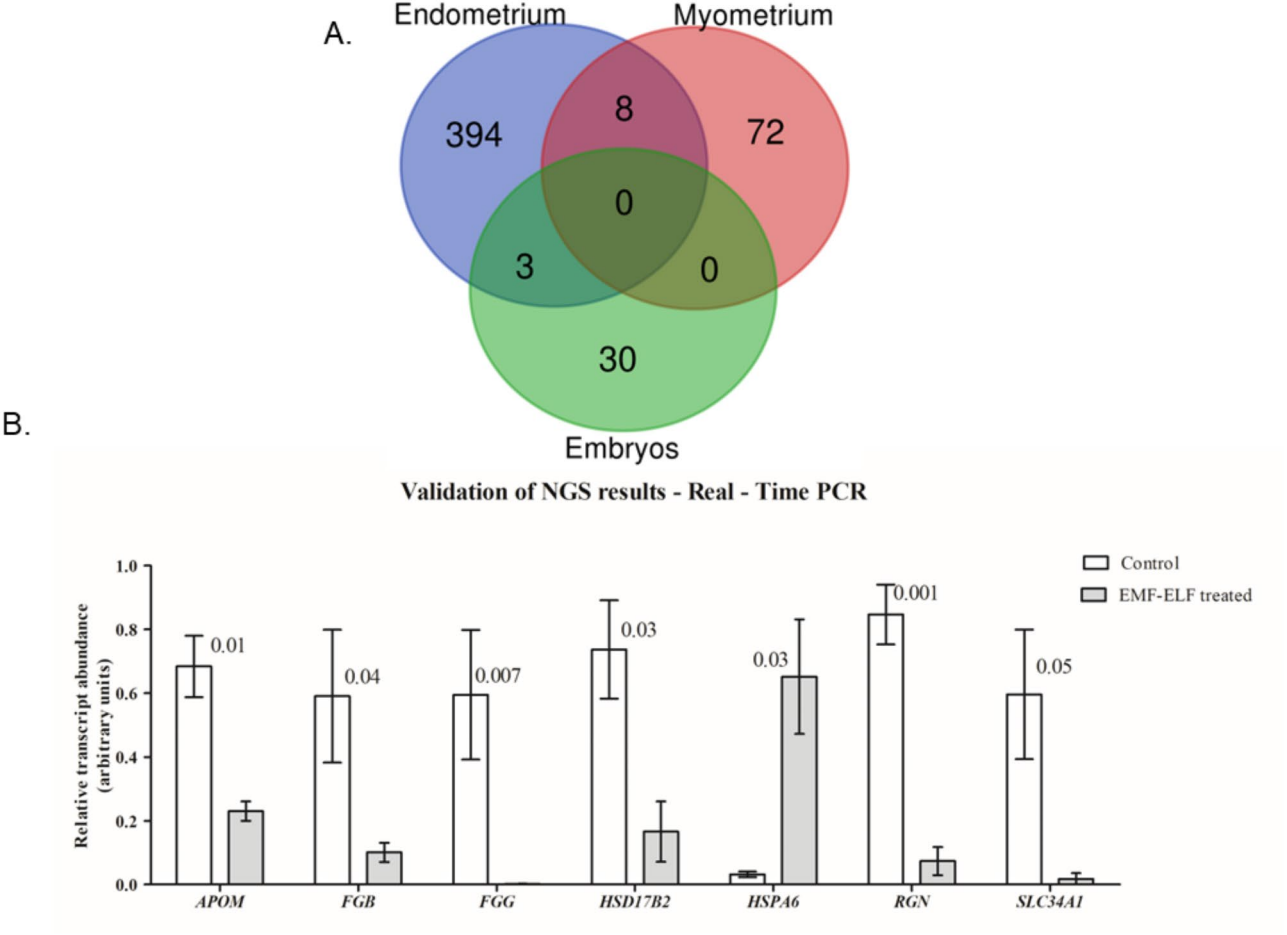
VENN diagram and real time validation results. (**A**) VENN diagram showing ELF-EMF-induced changes in the transcriptomes of the conceptuses, myometrium, and endometrium. (**B**) The expression profiles of selected mRNAs validated by real-time PCR within the NGS analysis of differentially expressed genes (DEGs). The data are presented as the means ± SEMs.

A comparison of both the up- and downregulated genes in ELF-EMF-treated conceptuses, the endometrium, and the myometrium revealed 3 DEGs common to the conceptuses and endometrium (*HPX, HSD17B2, PCK1*) and eight common to the endometrium and myometrium (Fig. 5A). There were no genes common to all three studied tissues. A summary of the ELF-EMF-related DEGs in the conceptuses, endometrium, and myometrium is presented in Supplementary Table S7.

### Validation of NGS results

The validation procedure elucidated that the relative mRNA transcript abundance of the selected DEGs (*APOM, FGB, FGG, HSD17B2, HSPA6, RGN, SLC34A1*) significantly differed in the conceptuses tissue treated in vitro with an ELF-EMF at 50 Hz when compared to the non-treated that one. The results of the real-time validation converged with the direction of changes (up- or downregulation) obtained as a result of NGS analysis (Supplementary Material S8). Thus, the validation procedure confirmed the consistency of the obtained NGS results. The expression profiles of mRNAs encoded by validated genes are presented in Fig. 5B.

### Effect of ELF-EMF on the global level of genomic DNA methylation in porcine conceptuses during the peri-implantation period

**Fig. 6:**
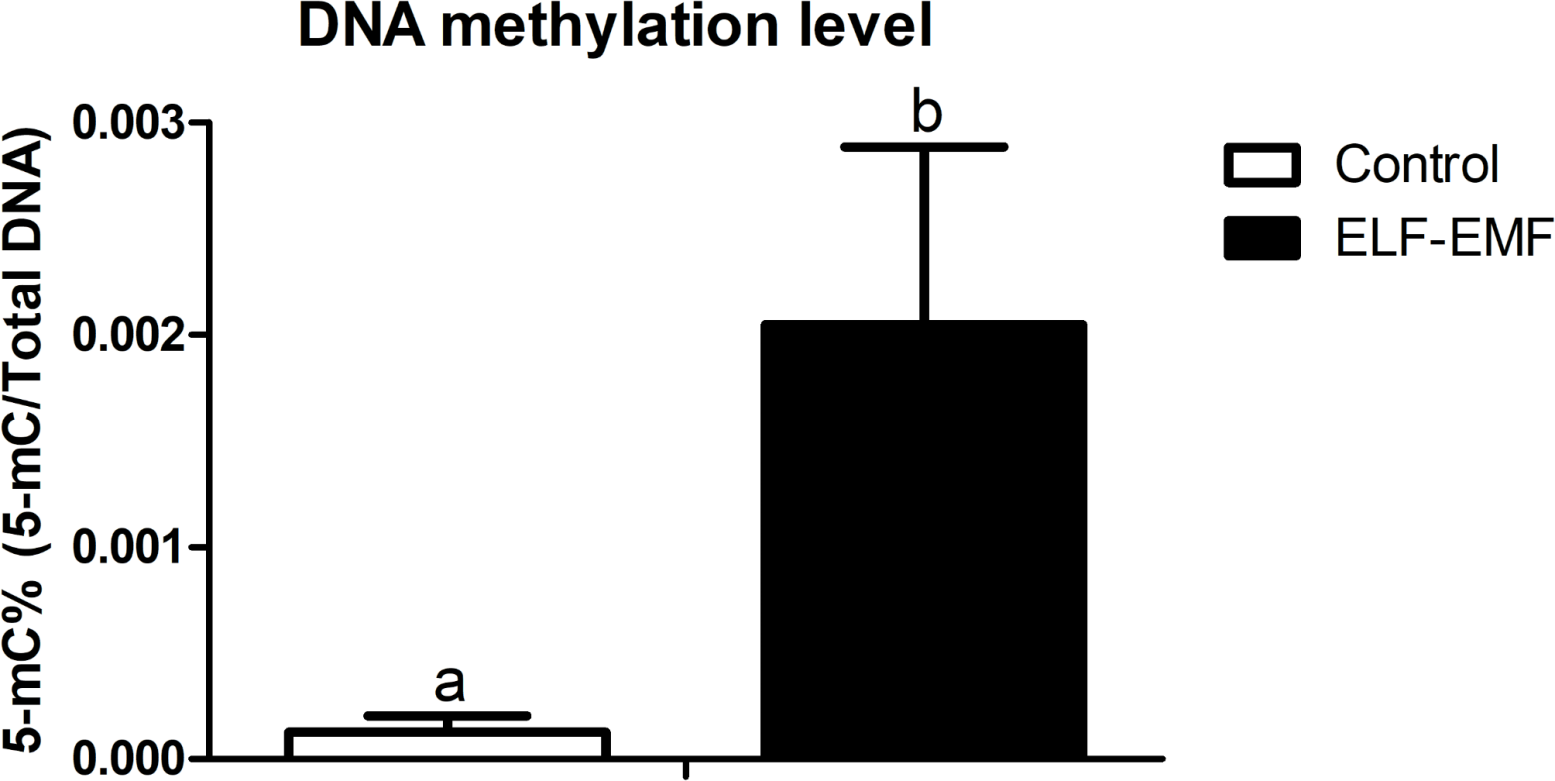
ELF-EMF- influence on genomic DNA methylation in porcine conceptuses. The level of global DNA methylation in control and ELF-EMF (50 Hz, 2 h, 8 mT)-treated conceptuses tissue of pigs during the peri-implantation period. Results are expressed as mean ± standard error of the mean (SEM). Different lower-case letters (a, b) indicate statistically significant differences (*P* ≤ 0.05).

The level of genomic DNA methylation in porcine conceptuses isolated during the peri-implantation period was significantly greater and increased 16.37 times in conceptuses treated with an ELF-EMF at 50 Hz than in embryos not treated with an ELF-EMF (Fig. 6, *P* ≤ 0.05).

### Quantitative methylation-specific PCR analysis of DNA methylation levels in conceptuses

**Fig. 7:**
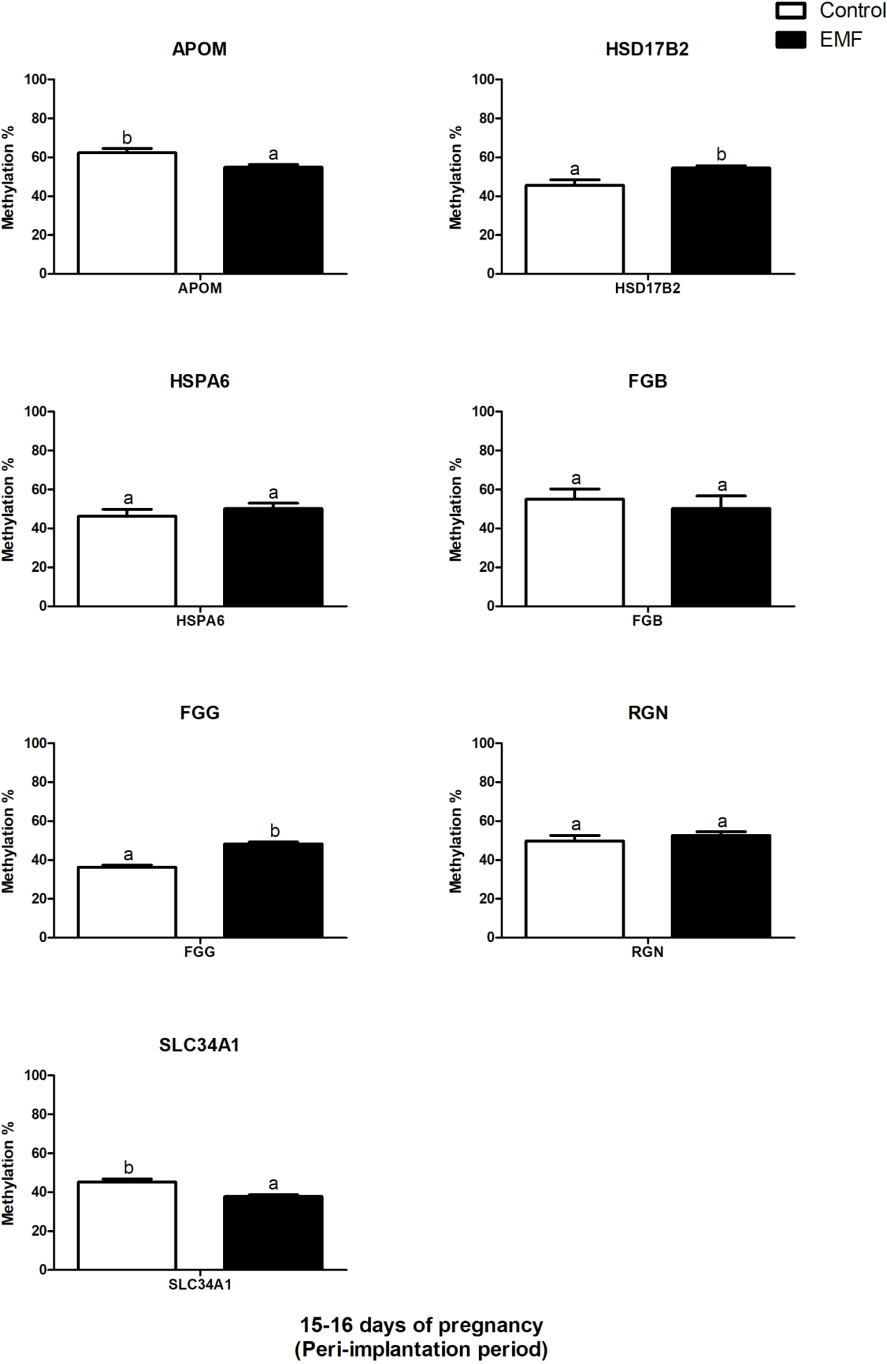
Percent methylation of genes with the altered expression in control and ELF-EMF (50 Hz, 2 h, 8 mT)-exposed embryo pigs during the peri-implantation period. Different lower-case letters (a, b) indicate statistically significant differences (*P* ≤ 0.05).

The percentage of DNA methylation based on the amplification dataset of selected genes in conceptuses is presented in Fig. 7. Compared with those in nonexposed conceptuses, the percentages of *APOM* and *SLC34A1* methylation in DNA from ELF-EMF-exposed conceptuses were significantly lower (*P* ≤ 0.05, Fig. 7). The percentage of DNA methylation in *FGG* and *HSD17B2* was significantly greater in conceptuses exposed to ELF-EMF than in control conceptuses (without ELF-EMF, *P* ≤ 0.05; Fig. 7). There was no significant difference in the percentage of *FGB, HSPA6,* or *RGN* DNA methylation in the ELF-EMF-treated conceptuses (*P* ≥ 0.05, Fig. 7).

### Effect of ELF-EMF on the amplification of methylated and unmethylated promoters of selected genes in conceptuses

**Fig. 8:**
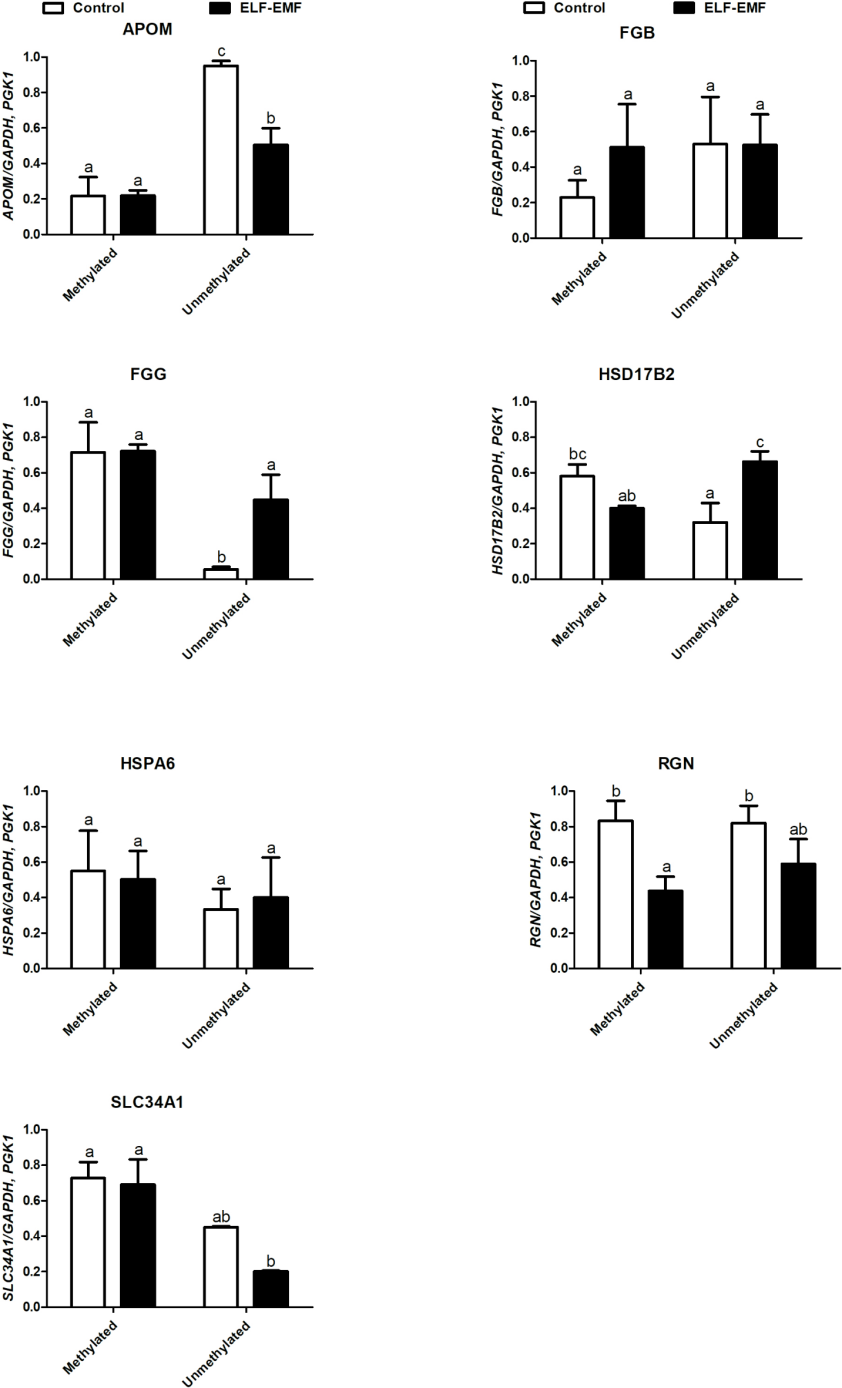
The level of amplification of methylated and unmethylated promoter regions. The level of amplification of methylated and unmethylated promoter regions of genes whose expression was altered in control and ELF-EMF (50 Hz, 2 h, 8 mT)-exposed embryo pigs during the peri-implantation period. Different lower-case letters (a, b) indicate statistically significant differences (*P* ≤ 0.05).

The effect of ELF-EMF on the amplification of methylated and unmethylated sequences of promoters of selected genes in conceptuses is presented in Fig. 8. The influence of ELF-EMF on the amplification of the methylated and unmethylated promoter regions of *FGB, HSPA6,* and *SLC34A1* was not observed (*P* ≥ 0.05, Fig. 8). The amplification of unmethylated promoter regions of *FGG* and *HSD17B2* was significantly increased in conceptuses exposed to the ELF-EMF (*P* ≤ 0.05, Fig. 8). Amplification of unmethylated promoter regions of *APOM* and methylated promoter regions of *RGN* in porcine conceptuses was significantly decreased by the ELF-EMF compared to control. In conceptuses non-exposed to the ELF-EMF, the methylated promoter regions were increased compared to the unmethylated promoter regions of *FGG* and *HSD17B2* (*P* ≤ 0.05, Fig. 8) and decreased compared to the unmethylated promoter regions of *APOM* (*P* ≤ 0.05, Fig. 8). In the ELF-EMF-treated conceptuses, methylated promoter region of *SLC34A1* was increased, whereas promoter region of *APOM* and *HSD17B* were decreased compared to unmethylated promoter region (Fig. 8).

## Discussion

The present study determined that short treatment (2 h) duration of ELF-EMF exposure affects the transcriptomic profile, alternative splicing and SNV occurrence, DNA methylation level, and the level of amplification of methylated and unmethylated variants in promoter regions of selected DEGs in conceptuses in vitro. The results revealed that the global DNA 5-mC methylation level increased in the response to ELF-EMF treatment. On the other hand, any differences in genes encoding the methyltransferases DNA—DNMT1 and DNMT3—proteins were observed. Therefore, it is possible that only the final outcome of the methylation process, expressed as an increase in the percentage of 5-mC in conceptuses exposed to ELF-EMF, was determined. The increase in 5-mC methylation of DNA is generally recognized to have a significant effect on gene silencing^32^. Therefore, the exposure of peri-implantation conceptuses to the ELF-EMF might result in changes connected with the 5-mC modifications. Similar alterations were also determined previously in porcine endometrium and myometrium exposed to the ELF-EMF^26,33^.

The results of transcriptomic analyses s revealed that 17 DEGs with the assigned gene names were downregulated in conceptuses exposed to the ELF-EMF. Although the pattern of DNA methylation in conceptuses during the peri-implantation period is established^34^, some data indicate that external factors, i.a.. maternal undernutrition during the peri-conceptional period or estradiol 17-β (E_2_) treatment, may affect the DNA methylation level^35–37^. From this perspective, the observed increased level of global DNA methylation in conceptuses may be the result ELF-EMF treatment. However, further studies should explore the changes in 5-mC methylation and demethylation mechanisms in porcine conceptuses exposed to ELF-EMF to determine the more in-depth mechanisms of global DNA methylation.

Interestingly, we have determined that three separate genes, i.e., FGA, FGB, and FGG, were downregulated and enriched in the KEGG:04610 *Complement and coagulation cascades pathway*, GO BP terms, whereas FGG enriched the *plasma membrane* and *cell periphery* GSEA terms in conceptuses exposed to the ELF-EMF. *FGA, FGG,* and *FGB* mRNA transcripts encode protein chains of soluble fibrinogen Aα-, fibrinogen Bβ-, and fibrinogen γ, respectively^38^. The presence of mRNA transcripts encoding fibrinogens was previously reported also in the peri-implantation equine^39,40^ and ovine conceptuses^41^. It has been highlighted that disrupted interactions between conceptus-derived fibrinogen and integrins may contribute to the interruption of conceptus mobility within the uterus^39^. Considering the known repressive effect of the ELF-EMF treatment on the abundance of all mRNA transcripts encoding fibrinogen proteins, it may be indicated that ELF-EMF treatment affects processes engaged in cell adhesion as well as those at work to ensure the mobility of porcine conceptuses during the peri-implantation period. Intriguingly, the process of DNA methylation was found to be a potential regulatory mechanism of fibrinogen synthesis in the equine endometrium and conceptuses^39^. In the present study, we provided evidence that the percentage of methylation of the *FGG* gene and its unmethylated promoter region were increased in porcine conceptuses exposed to the EMF-EMF. In contrast, we did not detect any impact of the ELF-EMF on the level of *FGB* methylation. Respecting that the methylation of DNA is recognized as a repressor of transcriptional activity, increased methylation level of *FGG* may be one of the mechanisms of its downregulated expression in porcine conceptuses exposed to ELF-EMF. On the other hand, alternative mechanisms may be likely involved in the control of *FGB* transcription. The further investigation is needed to clarify this point.

Notably, the exposure of conceptuses to the ELF-EMF resulted in the upregulation of the *SERPINE1* mRNA transcript, encoding the SERPINE1 protein which is associated with developmental processes and the prevention of early implantation^42^. In parallel, increased expression of PLG activator (PLAT) was previously observed in the porcine endometrium exposed to ELF-EMF^10^. Interestingly, both proteins, i.e., SERPINE1 and PLAT, regulate fibrinogen/fibrin homeostasis and are related to fibrinolysis process^43^. Respecting the important role of cell adhesion process in conceptus–endometrial communication during implantation, increased abundance of *SERPINE1* mRNA transcript in conceptuses *PLAT* mRNA transcript in the endometrium as the consequence of ELF-EMF exposure, may negatively affect the ability of the conceptuses adhesion to the endometrium during the peri-implantation period.

The results of the present study indicated that ELF-EMF treatment results in decreased expression of the *RGN* gene, encoding regucalcin which is necessary for calcium binding and acts as a suppressor of intracellular calcium signaling^44^. *RGNs* were enriched in the GO terms 0016787 *hydrolase activity* and GO term 0043167 *ion binding* in the GSEA. Interestingly, calcium ions play essential roles in the regulation of many physiological processes, including hormone secretion, the cell cycle, gene expression, neurotransmission, and muscle contraction^44,45^. Similarly, they are also important for the maintenance of fibrinogen structure and function^38^. Therefore, in addition, downregulatory effect of the ELF-EMF on genes encoding fibrinogen subunits, decreased *RGN* expression may affect the interactions between conceptuses and the endometrium. Importantly, the down-regulation in the *RGN* transcription coincides with the decreased amplification of the methylated promotor of this gene in ELF-EMF exposed conceptuses whereas the level of *RGN* gene methylation was unchanged. Taken together, these findings indicate that DNA methylation is unlikely to be the only mechanism responsible for the ELF-EMF-dependent decrease in *RGN* expression in porcine conceptuses.

In the present study, the down-regulation of *HSD17B2* expression in conceptuses treated in vitro with ELF-EMF was observed. It is known that the proper synthesis and release of conceptuses estrogens determines the proper activity of uterine tissues and creates intrauterine environment helpful to maintain of pregnancy. Any alterations in estradiol-17β synthesis and secretion are detrimental for porcine embryos and may affect embryo-maternal interactions during implantation and lead to pregnancy lost^46,47^. It is worth noting that the excessive exposure of porcine embryos to estrogens induces deleterious effects in their development^48^. Interestingly, the HSD17B2 knockout (KO) resulted in 70% embryonic lethality starting from embryonic day 11.5 in mice and 24% of HSD17B2KO mice that survive through the fetal period are born growth retarded^49^. Additionally, HSD17B2KO mice exhibit reduced placental size and its structural abnormalities. These findings document indisputable role of HSD17B2 in embryogenesis^49^.

Consistently, decreased expression of the *HSD17B2* mRNA transcript observed in the ELF-EMF-treated conceptuses was accompanied by increased level of *HSD17B2* methylation. Therefore, the amplification of the unmethylated promoter region of *HSD17B2* was also elevated while the methylated promoter region of *HSD17B2* was not changed in conceptuses exposed to the ELF-EMF suggesting that the ELF-EMF-induced DNA may be partially responsible for *HSD17B2* down-regulation in conceptuses. This notion indicates that the unmethylated promoter region of *HSD17B2* is more sensitive to the ELF-EMF exposure than methylated one. It cannot be excluded that the reason for these differences leads to different organizations of CpG islands structure^50^. Decreased abundance of *HSD17B2* mRNA transcript was also determined in the porcine endometrium exposed to the ELF-EMF in vitro^10^. We believe alterations in epigenetic modification of *HSD17B2* expression are of interest since the important role of HSD17B2 gene encoding 17β-hydroxysteroid dehydrogenase type 2 (17βHSD2) involved in the conversion of estradiol-17β (E2) to estrone (E1)^51^, and deactivation of androgens and activation of progestins^51^. The 17β-HSD2 plays the role in the modulation of active steroid hormone availability, including estrogens and androgens, and being defined as “antiandrogenic” and “antiestrogenic”^52^. Therefore, increased level of *HSD17B2* methylation and down-regulation of *HSD17B2* expression in porcine conceptuses exposed to the ELF-EMF may disturb the proper conversion of estradiol-17β (E2) to estrone (E1) and lead disturbed production of steroids in conceptuses and their concentration in intrauterine environment. This suggestion is justified because of the risky effect of the ELF-EMF radiation on steroidogenesis documented in porcine conceptuses in vitro^11^.

The results of the current study revealed that the exposure of conceptuses to ELF-EMF affects genes encoded lipoproteins with alterations in the GO:0034362 *low-density lipoprotein particle*, GO:0034364 *high-density lipoprotein particle*, GO:0034361 *very-low-density lipoprotein particle,* and GO:0034385 *triglyceride-rich plasma lipoprotein particl*e cellular component terms. All of the above-mentioned enriched GO terms are associated with the downregulation of the apolipoprotein M (*APOM*) gene. *APOM* encodes apolipoprotein, which is associated mainly with high-density lipoprotein (HDL) in plasma and, in a small proportion, with triglyceride-rich lipoproteins and low-density lipoproteins (LDLs)^53^. Apolipoprotein M (APOM) was detected in liver and kidney of mouse embryos from day 7.5 to 18.5 of embryonic development and liver and kidneys of human foetuses from months 3–5 and 5–9 of pregnancy, respectively. The *APOM* expression was also observed in the small intestine, skeletal muscle, and stomach at various stages of embryonic development^54^. The past study indicated the potential role of APOM in neural tube development in human foetuses^55^. At present, the physiological significance of *APOM* during embryogenesis in mammals is not fully understood, and the mechanism regulating *APOM* involvement in lipid and/or lipoprotein metabolism in porcine conceptuses needs further investigation. To date, *APOM* belongs to the lipocalin superfamily which has the ability to carry hydrophobic molecules, such as steroids and retinoids, in the blood plasma^56,57^. Additionally, the results of studies carried out in HepG2 cells have shown that E_2_ upregulates *APOM* expression through an ER-α-dependent pathway involving the ER-α binding element in the promoter of the *APOM* gene^58^. As observed in the current study decreased *APOM* expression was not accompanied by increased methylation but was instead accompanied by decreased amplification of the unmethylated promoter region. Such surprising evidence was also observed in the porcine endometrium and the myometrium exposed to ELF-EMF during the peri-implantation period^25,26^. Nowadays, we have no clear explanation for this finding. However, given that APOM is involved in the synthesis of HDL, LDL, and triglyceride-rich lipoproteins, it cannot be excluded that the observed downregulation of *APOM* expression in conceptuses exposed to the ELF-EMF may affect their metabolism and cholesterol, steroids and/or other bioactive hydrophobic molecules transfer.

Another interesting observation of the present study is the down-regulation of the *SLC34A1* gene in conceptuses exposed to the ELF-EMF. To our knowledge, this is the first report on *SLC34A1* expression in porcine conceptuses. *SLC34A1* encodes the sodium-dependent phosphate transporter 2A (NaPi-IIa) which is crucial for phosphate balance^59,60^. The specific role of SLC34A1in embryo development is not defined yet. However, it was documented that knockout mice lacking Npt2b (SLC34A2), the other type of phosphorus transporter, died shortly after implantation^61^. The results of the current study documented that the ELF-EMF exposure may affect the sodium-dependent phosphate transporter in the peri-implantation conceptuses. ELF-EMF-related alternations in *SLC34A1* may therefore disrupt phosphorus levels and negatively impact embryonic cell activity. Notably, phosphorus is essential for the proper cell signaling (phosphorylation), cells function (structure and activity of nucleic acids), cell membrane organization and energy metabolism (ADP and ATP)^62^. Therefore, any disturbances in the phosphorus milieu are likely to have negative consequences for embryogenesis. However, it should be noted that DNA methylation is not responsible for the decreased expression of *SLC34A1* observed in the response to ELF-EMF treatment, since in exposed conceptuses, the percentage of *SLC34A1* methylation was lower vs. control ones. Similarly, ELF-EMF does not affect the amplification of the unmethylated and methylated promoter regions of *SLC34A1.* The lack of association between global DNA methylation and the level of gene expression was also observed^63,64^.

The upregulation of *HSPA6* in porcine conceptuses exposed to the ELF-EMF was detected. The *HSPA6* encodes the member of antioxidant enzymes of the HSP70 family which is crucial for cellular homeostasis and antioxidation^65^. HSP70 proteins synthesized in conceptuses during their pre-implantation development are essential for decidualization, implantation, and placentation^66,67^. Inhibiting heat shock proteins impedes blastocyst growth and increases embryonic apoptosis, underscoring their importance in creating a conducive environment for implantation and preventing the pre-implantation embryo death^68^. Observed in the current study *HSPA6* upregulation indicates that the potential for antioxidant ability may be increased in conceptuses exposed to the ELF-EMF. Observed changes in *HSPA6* expression appeared to be independent of variations in DNA methylation, and neither amplification of the methylated nor unmethylated promoter regions changed in response to ELF-EMF radiation. A similar effect was observed in the case of other mRNA transcripts. Interestingly, changes in *HSPA6* expression were observed also in the response to stress conditions^69^. Indeed, pigs exposed to heat stress conditions during the peri-implantation period presented increased *HSPA6* expression in the endometrium, whereas this effect was not observed in females during the estrous cycle^69^. It is worth mentioning that the ELF-EMF ability to induce oxidative stress was also previously confirmed as the abundance of *NOS3* mRNA transcript was increased in the ELF-EMF-exposed endometrium^10^. It cannot be excluded that the ELF-EMF-induced increase in *HSPA6* expression may serve as a potentially protective mechanism to ensure uninterrupted processes involved in communication between the d conceptuses and uterine tissues.

In the current study we focused on the SNVs in genes annotated to *EGFR downregulation*, i.e., *PTPN12, EPN1,* and *EGFR,* and missense changes, which may be important from a reproductive success point of view. The gene *PTPN12* (C > T, indicating two changes, an intron variant and a synonymous variant), encodes protein-tyrosine phosphatase (PTP)-PEST (PTPN12)^70^. This protein expression is essential in processes involved in protein‒protein interactions, the regulation of apoptosis, normal vascular development, adhesion, and the migration of endothelial cells^70^. Moreover, PTPN12 plays a key role in normal embryonic development and viability since the lethality in PTP-PEST-deficient mouse embryos was observed^71^. The second mentioned above gene, *EPN1* (C > T, upstream gene variant)*,* encodes epsin 1, which regulates endocytic process and the exit of embryonic stem cells from the pluripotent stage to their future differentiation^72^. The third is the *EGFR* gene (C > T, 3’ prime UTR variant) which encodes the epidermal growth factor receptor (EGFR). The EGF acts via EGFR and in porcine embryos during the peri-implantation period induces multiple cell signaling pathways that are critical for the proliferation, migration and survival of trophectoderm cells^73^. These processes are therefore likely to be altered in ELF-EMF-treated conceptuses. Notably, the same alteration in the EGFR gene was found in ELF-EMF-treated porcine myometrium^9^, highlighting the potential of ELF-EMF for regulation of EGFR-mediated processes in the uterus of pigs including mechanisms of embryo-maternal communication during the peri-implantation period.

Finally, the present study identified the SNVs induced by the ELF-EMF in conceptuses. It was documented that the ELF-EMF affects missense substitutions in the lipolysis-stimulated receptor (*LSR),* angiogenin (ANG)-ribonuclease inhibitor synthesis (RNH1)*,* transducin (beta)-like 3 *(TBL3),* and LIM And SH3 Protein 1 (*LASP1)* genes. Observed changes could lead to alterations in the encoded protein sequence, folding, and structure, and may detrimentally affect the developmental potential of the conceptuses. For example, the lipolysis-stimulated receptor (LSR), encoded by the *LSR*, is responsible for apolipoprotein B/E-containing lipoproteins. The lack of the LSR in LSR−/− murine embryos caused their lethality between 12.5 and 14.5 days of gestation^74^. The *RNH1* gene encodes angiogenin (ANG)-ribonuclease inhibitor synthesis, which is necessary to control the translation machinery^75^*.* Additionally, in zebrafish embryos, transducin (beta)-like 3 (*TBL3*) plays a tissue-specific role by regulating the cell cycle rate during embryo development^76^. The *LASP1* is a key factor in trophoblast invasion and migration^77^. Considering these findings, it is worth highlighting that SNVs induced by ELF-EMF radiation were identified in genes that are crucial for conceptuses development during the peri-implantation period. We believe that research on SNVs/SNPs occurring in conceptuses as well as in female reproductive tissues exposed to the ELF-EMF will be crucial for exploring the basis of reproductive disorders to understand the reasons for early pregnancy failure.

The current study may have significant translational implications for human reproductive health, including the aspects of cellular homeostasis, conceptuses development, and their attachment. However, before considering final translational implications, it is crucial to conduct the next comprehensive studies on mechanistic pathways activated by the ELF-EMF or pathways inhibition and/or overexpression, and protein levels of DEGs. Such investigations will provide a solid foundation for understanding the underlying biological processes and increase the reliability of the potential translational outcomes to develop a strategy of conceptuses protection against the ELM-EMF. Moreover, the insights gained from the current research may contribute in the future to the development of evidence-based guidelines for the ELF-EMF exposure limits, especially for women with planned pregnancy and early pregnant women and in healthcare settings. Although, it is crucial to acknowledge the limitations of the current study when considering its translational potential. The use of an in vitro porcine conceptuses model, while allowing for controlled experiments, may not fully replicate the in vivo environment during early pregnancy. Therefore, additional in vivo studies are called to provide specific implications including the potentially risky impact associated with the ELF-EMF. The future research directions should include in vivo validation and the study of long-term exposure effects to elucidate its impact on fertility and pregnancy outcomes and to enhance ecological validity. We are aware that species-specific differences between porcine and human embryos development could limit the direct applicability of obtained findings to human reproductive health. To address these limitations future research should be aimed to translate the implications.

In conclusion, although humans and animal are exposed to an increasing number of artificial ELF-EMF sources for many years, the full extent of the ELF-EMF impact on reproduction is still unknown. Notably, the short-treatment duration of ELF-EMF exposure at the frequency of 50 Hz induces changes in transcriptional mechanisms (SNVs and AS) and affects the level of DNA methylation in conceptuses during the peri-implantation period (Supplementary Material S9). The ELF-EMF exposure disrupts the level of expression of genes involved in cellular homeostasis, conceptuses attachment and their development. Future studies should explain the potential translational implications of obtained findings for human reproductive health, particularly for those exposed to ELF-EMF in industrial or medical contexts. The future research directions should include in vivo validation of the ELF-EMF effects and determination of long-term ELF-EMF exposure effects to elucidate its impact on female fertility and pregnancy development. The in vivo study is called to provide more specific implications including elucidating the ELF-EMF long-term effects on mammalian conceptuses development and determining the reproductive risk in future generations associated with the ELF-EMF radiation nowadays.

## Materials and methods

### Ethics statement

The study was carried out in compliance with the ARRIVE guidelines^78^, and all methods were carried out following relevant guidelines and regulations where appropriate. Ethical review and approval were waived for this study because all the experiments were conducted on animal tissues collected post-mortem during regular economic slaughter provided in a professional slaughterhouse. The use of animals followed the Act of the 15th of January 2015 on the Protection of Animals Used for Scientific or Educational Purposes and Directive 2010/63/EU of the European Parliament and the Council of 22nd of September 2010 on the protection of animals used for scientific purposes.

### Animals, collection of conceptuses, and ELF-EMF treatment

All experimental procedures were performed on porcine conceptuses collected from pigs during the peri-implantation period (days 15–16 of pregnancy) and not exposed or exposed in vitro to the ELF-EMF at 50 Hz. The procedures for pig insemination, collection of conceptuses, preincubation, and incubation in ELF-EMF conditions were described previously by Franczak et al.^11^. In brief, conceptuses were obtained from pigs (Sus scrofa domestica L., Polish Landrace × Great White Polish, weighing 95–110 kg; n = 4) on days 15–16 of pregnancy. To collect conceptuses, the uterus was flushed three times with PBS mixed with M199 at a ratio of 1:1. The conceptuses were examined to confirm their morphology typical to a peri-implantation porcine conceptuses and to confirm the stage of pregnancy^79,80^. The conceptuses were then centrifuged (Centrifuge 5804 R; Eppendorf, Hamburg, Germany) at 45 × g for 8 min at room temperature to remove washing media and uterine flushings. Conceptuses collected from each pig were pooled and considered as one biological replicate (n). The collected conceptuses were not separated into embryonic disc and extraembryonic membranes. A was previously described by Franczak et al.^11^, all flushed conceptuses were transferred to 120 mm diameter Petri dishes and covered with 15 mL of M199 (Sigma Aldrich, Darmstadt, Germany) supplemented with 5% newborn calf serum (NCS; Sigma Aldrich, Darmstadt, Germany; N4762-100ML) and 1% commercial antibiotic–antimycotic solution and were first preincubated for 6 h at 37 °C, 85% humidity, 5% CO_2_, and 95% air. Following the pre-incubation, conceptuses were cut into 100 mg ± 5% fragments and randomly divided into control group (without ELF-EMF, n = 4 biological replicates) or exposed to an ELF-EMF group at a frequency of 50 Hz, 8 mT for 2 h using an Astar generator and dedicated applicators (Magneris Astar, Bielsko-Biala, Poland) and cultured separately under the same atmospheric conditions. A description of the EMF exposure system and the time and frequency choices and rationale were previously described in detail^11,14–17^. Conceptuses, considered controls were not exposed to the ELF-EMF. The temperature was monitored during the entire in vitro incubation period to exclude any negative thermal effects on the in vitro culture. After incubation, the conceptuses fragments were collected, washed in phosphate-buffered saline, dehydrated with a sterile paper filter, frozen in liquid nitrogen (− 196 °C), and stored at − 80 °C for further analyses.

### RNA isolation

TRI Reagent (Sigma Aldrich, St. Louis, MA, USA) and RNeasy Mini Kit (Qiagen, Valencia, CA, USA) were used for total RNA extraction from conceptuses following custom protocols as described previously by Drzewiecka et al.^9^ with minor modifications. Briefly, a 10 mg conceptuses mass was mixed well by pipetting and briefly vortexing with 500 µl of TRI Reagent. Next, the samples were incubated on ice for 60 min and briefly mixed by vortexing every 10 min until full digestion was completed. Next, the RNA fraction was separated using 150 µl of ice-cold chloroform and mixed up with 200 µl of 70% ethanol, which was subsequently frosted to -20 °C. The RNA-ethanol samples were subsequently transferred to an RNeasy Mini spin column (Qiagen, Valencia, CA, USA) and proceeded according to the manufacturer’s protocol (Qiagen, Valencia, CA, USA). The RNA was eluted with 80 µl of DEPC-treated water and instantly measured spectrophotometrically for initial determination of the purity (optical density, OD, A260/A280) and concentration (ng/µL) of the obtained RNA. Only samples with ODs of 1.8–2.0 and concentrations > 500 ng/µL were subjected to further next-generation sequencing (NGS). Next, aliquots of the obtained RNA were prepared for measurement of the RNA integrity number (RIN; 28 S/18 S ratio) using 2100 Bioanalyzer with RNA 6000 Nano LabChip kit (Agilent Technologies, Santa Clara, CA, USA), NGS (Illumina Macrogen, Seoul, North Korea), and validation of the NGS results. Prepared aliquots were frozen at − 80 °C, and each aliquot was handled only once through the freezing‒refreezing cycle.

### Construction and sequencing of complementary DNA (cDNA) libraries – next-generation sequencing

For the construction and sequencing of cDNA libraries, isolated RNA aliquots with RNA integrity number (RIN) ≥ 8 and a concentration > 20 ng/µL were used. Preparation of cDNA libraries and sequencing were carried out by an outsourcing company (Macrogen, Seoul, North Korea) using the Illumina NovaSeq 6000 System (Illumina, San Diego, CA, USA). In brief, the TruSeq mRNA stranded libraries were prepared, and sequencing was performed by selecting the following run configurations: 2 × 150 bp and a throughput of 40 M paired reads per sample. The raw NGS data were deposited in the European Nucleotide Archive (ENA) under accession No. PRJEB60584 and were used for subsequent transcriptome profiling, bioinformatic analysis of gene expression, alternative splicing analysis, and single nucleotide variant description.

### Transcriptome profiling

Transcriptome profiling was performed for samples from the control (*n* = 3) and experimental (*n* = 3) RNA-seq libraries. Illumina adapter sequences and low-quality reads were removed using Trimmomatic software *v.* 0.38 (https://github.com/usadellab/Trimmomatic)^81^. Reads with an average PHRED score < 20 at the 3’ end and within the 10-base pair sliding window were discarded. The raw and trimmed paired-end reads were evaluated with FASTQC software *v.* 0.11.7^82^. The remaining paired-end reads were aligned to the pig reference genome with ENSEMBL annotation (Sus_scrofa.Sscrofa11.1.98) using the STAR *v.*2.7.3 mapper (https://github.com/alexdobin/STAR)^83^ and StringTie *v.* 1.3.3 (https://github.com/gpertea/stringtie)^84^ pipelines. Count values obtained within the spliced transcripts alignment to a reference (STAR) method (quantMode GeneCounts) were assigned to the transcriptionally active regions (TARs). Porcine TARs were divided into protein-coding genes and uncovered regulatory region groups. Before ELF-EMF and control sample comparisons, TARs with counts per million (cpm) < 1 in at least 3 samples were filtered out. The differential expression of genes was estimated by fitting a negative binomial distribution model and applying a shrinkage procedure to improve accuracy in DESeq2 v.1.44.0 (https://github.com/thelovelab/DESeq2) software^85^. Transcripts with an adjusted* p* value < 0.05 and an absolute value of logarithmic fold change (logFC) > 1 were further defined as differentially expressed TARs (DE-TARs). The DE-TARs were divided into DEGs and noncoding RNAs (ncRNAs).

Next, gene ontology (GO) enrichment and gene set enrichment (GSEA) analyses were carried out for functional annotation using clusterProfiler v.3.10.0 (https://guangchuangyu.github.io/software/clusterProfiler/)^86^ R package and g:Profiler v.0.2.3 (https://biit.cs.ut.ee/gprofiler/gost) tool^87^. DEGs were grouped according to three GO categories, REACTOME annotation, signaling pathway signatures in the Kyoto Encyclopedia of Genes and Genomes (KEGG) (https://www.genome.jp/kegg/)^88,89^, and human phenotype ontology (HP) databases were enriched using g:Profiler^87^. The enrichment classification was conducted at an adjusted *p* value < 0.05. The final consensus DE-TARs were visualized in MA, Volcano, and heatmap plots with gplots v.3.1.3.1 (https://github.com/talgalili/gplots), ComplexHeatmap v. 2.20.0 (https://github.com/jokergoo/ComplexHeatmap), the GOplot v.1.0.2 (https://github.com/wencke/wencke.github.io) Bioconductor packages, and a custom script in R.

### RNA editing site prediction

To determine the potential RNA editing sites with differences in the allele fraction between the ELF-EMF-treated and control conceptuses samples, the multiple rMATS-DVR v.1.0 (https://github.com/Xinglab/rMATS-DVR)^90^ and R Bioconductor pipelines were used. Briefly, binary alignment map (BAM) files of the saved mapped RNA-seq reads were recalibrated by the Picard tool (http://broadinstitute.github.io/picard). Second, single nucleotide variant (SNV) calling analysis was accomplished using rMATS-DVR and the Genome Analysis Tool Kit (GATK) v.3 (https://github.com/broadgsa/gatk)^91^. Next, a custom R script was used to filter out low-quality variants. SNVs were filtered out according to the GATK standard parameters: total depth of base coverage < 10; RMSMappingQuality < 40; QualitybyDepth < 2; MappingQualityRankSum <  − 12.5; and ReadPosRankSum <  − 8. Using ENSEMBL VCF and GTF files, single nucleotide polymorphism (SNP) annotations and gene locations were assigned to the significant variants. The reference and alternative allele frequencies (AAFs) of the identified SNVs were calculated and compared with python rMATS-DVR scripts.

High-quality SNVs with alternative allele occurrence in at least half of the RNA-seq samples were used for downstream analysis. Afterward, from further identification of the RNA editing variations, elimination of the SNVs positioned in the vicinity of the spliced junction sites within bidirectional genes and pseudogenes was performed. After filtration, possible candidates for RNA editing with no SNP annotation (rs ID) were divided into canonical (A-to-I and C-to-U) and noncanonical (all other possible base substitutions) substitutions. The AAF changes (∆AAF > 0.1; FDR < 0.001) between the ELF-EMF-exposed and control samples were estimated for canonical RNA editing substitutions only. All the RNA editing candidates were interpreted via the variant effect predictor (VEP) (https://www.ensembl.org/info/docs/tools/vep/index.html)^92^ and plotted via Circos v.0.69.9 (https://circos.ca/)^93^.

### Differential alternative splicing (AS) events

To predict alternative splicing differences, a replicate multivariate analysis of transcript splicing (rMATS v.3.2.5) (https://github.com/Xinglab/rmats-turbo)^94^ was used. For the calculation of all AS events, the percent spliced-in (PSI) value and trimmed paired-end reads were applied. The remapping BAM results of the ELF-EMF-treated and control samples were compared and statistically tested (FDR < 0.05) to obtain the differential AS events (ELF-EMF-treated vs. control) with ΔPSI > 0.1. The AS events were divided into five types: A5SS, A3SS, MXE, RI, and SE. Additionally, to create a Sankey plot linking differentially expressed genes (DEGs), alternative splicing (AS) events, and single nucleotide variant (SNV) expression, the ggalluvial v.0.12.5^95^ (https://github.com/corybrunson/ggalluvial) and ggplot2 v.3.5.1 (https://github.com/tidyverse/ggplot2)^96^ libraries from R Bioconductor were utilized.

### Comparison of the transcriptome profiles of the ELF-EMF-treated endometrium, myometrium, and conceptuses

Alterations in the transcriptome of ELF-EMF-treated conceptuses were compared with those in the ELF-EMF-treated endometrium and myometrium published previously by Drzewiecka et al.^9^ and Kozlowska et al.^10^. The lists of DEGs were used for the preparation of VENN analysis using a commercially available online tool (http://bioinformatics.psb.ugent.be/webtools/Venn/).

### NGS validation procedure

For validation of the NGS results, analysis of the relative mRNA transcript abundance (real-time PCR) of seven selected DEGs in conceptuses tissue that was not treated or treated in vitro with ELF-EMF at 50 Hz was performed. The selection of DEGs for validation was grounded in their contribution to embryonic development or the regulation of reproductive processes. The selected DEGs encode *APOM*, *FGB*, *FGG*, (*HSPA6*, *HSD17B2*, *SLC34A1* and *RGN*. The amplification of selected DEGs mRNA was conducted using TaqMan™ RNA-to-CT™ 1-Step Kit, and specific, predesigned TaqMan™ probes are presented in Table 1 (both, Thermo Fisher Scientific, Waltham, MA, USA). The amplification was conducted in an AriaMX system (Agilent Technologies, Santa Clara, California, USA) in a 10 µl total reaction volume using 4 pg/µl total RNA. Each sample was analyzed in duplicate. The analysis was conducted following the general guidelines for gene expression analysis^97^ and the standard manufacturer’s protocol. The relative abundance of the mRNA transcript abundance was determined using the ΔΔCt method. Statistical significance was assessed using a t-test within the Statistica software (version 13.3, StatSoft Inc., Tulsa, OK, USA, http://www.statsoft.pl).

**Table 1:** Taq Man Probes used in the validation of the NGS results experiment.

Gene Symbol	Assay ID
Differentially expressed genes	
*APOM*	Ss03392166_m1
*FGB*	Ss04328981_g1
*FGG*	Ss04329014_s1
*HSD17B2*	Ss04245816_m1
*HSPA6*	Ss03387784_u1
*RGN*	Ss03380996_u1
*SLC34A1*	Ss06920446_m1
Reference genes	
*ACTB*	Ss03376081_u1
*GAPDH*	Ss03374854_g1

### DNA isolation and DNA bisulfite conversion

Genomic DNA (gDNA) was isolated from 10 mg of conceptuses after in vitro culture using the ReliaPrep™ gDNA Tissue Miniprep System kit (Promega, Madison, Wisconsin, USA) following the manufacturer’s protocol. The details of the gDNA isolation process were described previously^25,26^. The quality and quantity of the isolated gDNA were examined spectrophotometrically using Infinite 200 M PRO (TECAN, Zürich, Switzerland). Only high-quality gDNA templates (500 ng/20 µL) were used for bisulfite conversion and quantification of DNA methylation.

DNA conversion was performed using the EpiJET Bisulfite Conversion Kit (Thermo Fisher Scientific, Waltham, MA, USA) according to the manufacturer’s procedure (protocol A). The rule of DNA bisulfite conversion was previously described in detail by Zglejc-Waszak et al.^35^. In brief, 500 ng of isolated conceptuses gDNA (20 µL of total volume) was mixed up with 120 µL of Modification Reagent solution. The samples were placed in a thermocycler at 98 °C for 10 min and at 60 °C for 150 min. After conversion, the converted DNA was purified using a Binding Buffer and DNA Purification Micro Column according to the manufacturer’s instructions, which included the following steps: addition of the mixture to the column, centrifugation, washing the column with linked DNA, desulfonation, washing again, and elution with 10 µl of elution buffer. To obtain the appropriate amount of converted DNA, four conversions per sample were performed. The converted DNA was measured spectrophotometrically (absorbance at 260 nm = 1.0) and stored at –20 °C to prevent DNA degradation.

### Quantification of global level of genomic DNA methylation in conceptuses

The MethylFlash Methylated DNA 5-mC Quantification Kit (EpiGentek, Farmingdale, NY, USA) was used to measure absolute DNA methylation by estimating the level of 5-methylcytosine (5-mC) in gDNA samples^33^. The analysis was performed according to the manufacturer’s procedure. In brief, the appropriate wells of the plate were filled with 80 µl of ME2 binding solution, ME3 solution (negative control), standard sample (diluted ME4 to 5 ng/µl), or 100 ng of DNA sample (each sample was analyzed twice for accuracy). Next, to define the well, ME2 was added. The plate was shaken and incubated at 37 °C for 90 min to bind the DNA. After binding, the plate was washed, and following capture, antibodies (ME5 solution) were added. The plate was incubated for the next 60 min at RT. Subsequently, detection antibodies (ME6, 30 min) and a solution for enhancer detection (ME7, 30 min) were added. Next, for the detection of the signal, ME8 (Developer Solution) and ME9 (Stop Solution) were added. After incubation, the signal was detected with excitation set at 530 nm and emission set at 590 nm using a Tecan microplate reader (Zürich, Switzerland). The quantification of the relative methylation level was performed according to the manufacturer’s formula and is presented as a percentage of 5-mC in total DNA isolated from conceptuses exposed and not exposed to ELF-EMF. Statistical analysis (Statistica software version 13.3, StatSoft Inc., Tulsa, OK, USA, http://www.statsoft.pl) was performed using t-test for independent groups and the significant difference between control and ELF-EMF-treated samples was considered with *P* < 0.05.

### Quantitative methylation-specific PCR

The qMS-PCR was performed for seven selected genes with altered expression (DEGs) in the conceptuses. The genes were selected on the basis of their function and altered expression because of ELF-EMF exposure. The specific primers for the unmethylated and methylated sequences were designed for the gene promoter regions containing the CpG islands of *APOM, FGB, FGG, HSD17B2, HSPA6, RGN,* and *SLC34A1* according to the procedures described in Zglejc and co-workers^35^, using MethPrimer v.2.0 online tool (http://www.urogene.org/cgi-bin/methprimer2/MethPrimer.cgi); and checked with OligoCalculator online tools (http://biotools.nubic.northwestern.edu/OligoCalc.html) and mFold (http://www.unafold.org/). The sequence reference genes (glyceraldehyde 3-phosphate dehydrogenase, GAPDH; phosphoglycerate kinase 1, PGK1) were previously designed^35^. In brief, to perform methylation-specific qPCR, 10 ng of converted DNA, 12.5 µl of SYBR® Green PCR Master Mix, specific primers, and RNase-free water were added to a total volume of 25 µl. Each sample was analyzed in duplicate. qPCR was performed with an ARIA MX apparatus (Agilent Technologies, Santa Clara, CA, USA). The concentrations of the primers, the thermal conditions of the reactions and the cycle numbers are presented in Table 2. The degree of methylation was calculated using the formula Cmeth = 100/[1 + 2(M − UM)]%, where M represents the amplification (2^-ΔΔ^ Ct) of the methylated sequence and UM represents the amplification (2^-ΔΔ^ Ct) of the unmethylated sequence. To compare amplification of M and UM promoter regions of selected DEGs induced by ELF-EMF in the conceptuses, two-way ANOVA with Fisher’s least significant difference (LSD) post hoc test (Statistica software version 13.3 StatSoft Inc., Tulsa, OK, USA, http://www.statsoft.pl) was used. Only analyses with p-values ≤ 0.05 were considered statistically significant.

**Table 2:** Primers used for quantitative methylation-specific PCR.

Gene symbol (official)/reference sequences		Primer sequences 5’ → 3’ (start nt–stop nt)	Amplicon length, nt	Primer annealing (°C)	Primer’s efficiency (%)	References
*APOM* NM_001040640.1	M	F: GTTTAGGGATAACGCGAGAGCGAGG (90–114) R: CTCGAATCCGTCGTAACTTAAACGA (200–176)	111	50	75.84	This study
	UM	F: AAGTTTAGGGATAATGTGAGAGTGAGG (88–114) R: TCTCAAATCCATCATAACTTAAACAAAT (201–174)	114	60	87.38	
*FGB* NM_001244113.1	M	F: TATAGATGACGGATAGATAAATACGGGT (677–704) R: AAATTCTTAAAAACCTCCCATACGACA (983–957)	307	65	85.17	This study
	UM	F: TATAGATGATGGATAGATAAATATGGG (677–703) R: AATTCTTAAAAACCTCCCATACAACA (982–957)	306	50	85.76	
*FGG* NM_001244524.1	M	F: TGGAAAAAGTTTATTGTAAATCGAGA (987–1012) R: TACCATACTATACATTATATCCCCGAAA (1097–1070)	111	60	89.33	This study
	UM	F: GGAAAAAGTTTATTGTAAATTGAG (988–1011) R: ACCATACTATACATTATATCCCCAAAA (1096–1070)	109	60	82.26	
*HSD17B2* XM_005670066.3	M	F: GTTAGGAAGAAAAGGTAGGGTGGTAGCGAT (560–589) R: TTTCCCCCTTTAAAACTCCTCCCCC (977–953)	458	50	91.26	Wydorski et al. 2023
	UM	F: GTTAGGAAGAAAAGGTAGGGTGGTAGTGAT (560–589) R: TTTCCCCCTTTAAAACTCCTCCCCC (977–953)	458	50	89.14	
*HSPA6* NM_001123127.1	M	F: ATTGACGTGTTAGGGCGATT (882–901) R: TAAAACTAAAACTAAACTAATTCTCGAT (982–955)	101	60	87.97	This study
	UM	F: GATATTGATGTGTTAGGGTGA (879–899) R: ACCTTAAAACTAAAACTAAACTAATTCTCA (986–957)	108	60	84.61	
*RGN* NM_001077220.1	M	F: GGTTCGGGTTGGAGGTAACGTA (622–643) R: ACTTCTACGAAAACGCGACG (798–779)	177	60	89.24	This study
	UM	F: GGTTTGGGTTGGAGGTAATGTAA (622–644) R: TCTACCCCAACTTCTACAAAAACACAACAC (807–778)	186	60	89.18	
*SLC34A1* NM_001044623.1	M	F: TTTATTGTGGAGTAGCGGAA (338–357) R: ACTAAAAATCTAATAACAACTATAACCGCC (529–500)	192	50	83.10	This study
	UM	F: TTTATTGTGGAGTAGTGGAA (338–357) R: AAATCTAATAACAACTATAACCACCA (524–499)	187	60	86.32	
Reference genes						
*GAPDH* NM_001206359.1		F: AGGTTGTGGGTAAGGTTATT (738–759) R: CCTACTTCACCACCTTCTTAAT (866–888)	150	60	86.34	Franczak et al.^11^, Wydorski et al.^25^
*PGK1* AY677198.1		F: GTGGATGGGTTTGGATTGT (1017–1036) R: CTTTCACCACCTCATCCATAAA (1150–1172)	155	60	87.89	

M, methylated sequence; UM, unmethylated sequence.

## Electronic supplementary material

Below is the link to the electronic supplementary material.

Supplementary Material 1

Supplementary Material 2A

Supplementary Material 2B

Supplementary Material S3

Supplementary Material S4

Supplementary Material S5

Supplementary Material S6

Supplementary Material S7

Supplementary Material S8

Supplementary Material S9

Supplementary Material S10

## Data Availability

All the data are incorporated into the article and as the online supplementary materials. Raw data are available in the European Nucleotide Archive (ENA) under accession No. PRJEB60584.

## Abbreviations

5-mC: 5-Methylcytosine
A3SS: Alternative 3′ splice site
A5SS: Alternative 5′ splice site
AAFs: Alternative allele frequencies
APOM: Apolipoprotein M
AS: Alternative splicing
BAM: Binary alignment map
BP: Biological process
CC: Cellular component
cDNA: Complementary DNA
cpm: Counts per million
DAS: Differentially alternative splicing
DEGs: Differentially expressed genes
E_2_: Estradiol 17-β
ELF-EMF: Extreme low-frequency electromagnetic field
ENA: European Nucleotide Archive
FGB: Fibrinogen beta chain
FGG: Fibrinogen gamma chain
gDNA: Genomic DNA
GO: Gene ontology
GSEA: Gene set enrichment analysis
HP: Human phenotype ontology
HSD17B2: Hydroxysteroid 17-beta dehydrogenase 2
HSPA6: Heat shock protein family A (Hsp70) member 6
KEGG: Kyoto Encyclopedia of Genes and Genomes
LSR: Lipolysis stimulated receptor
MXE: Mutually exclusive exons
ncRNAs: Noncoding RNAs
NGS: Next-generation sequencing
OD: Optical density
PLAT: PLG activator
PSI: Percent spliced-in
PTPN12: Protein-tyrosine phosphatase (PTP)-PEST
RGN: Regucalcin
RI: Retention intron
RIN: RNA integrity number
SE: Skipping exon
SLC34A1: Solute carrier family 34 member 1
SNP: Single nucleotide polymorphism
SNV: Single nucleotide variant
STAR: Spliced transcripts alignment to a reference
TARs: Transcriptionally active regions
