# Supplementary figures and images for: Effect of electromagnetic field radiation on transcriptomic profile and DNA methylation level in pig conceptuses during the peri-implantation period
Source: Sci Rep. 2025 Apr 23;15:14025. doi: 10.1038/s41598-025-98918-9 (PMC12019412; doi:10.1038/s41598-025-98918-9)

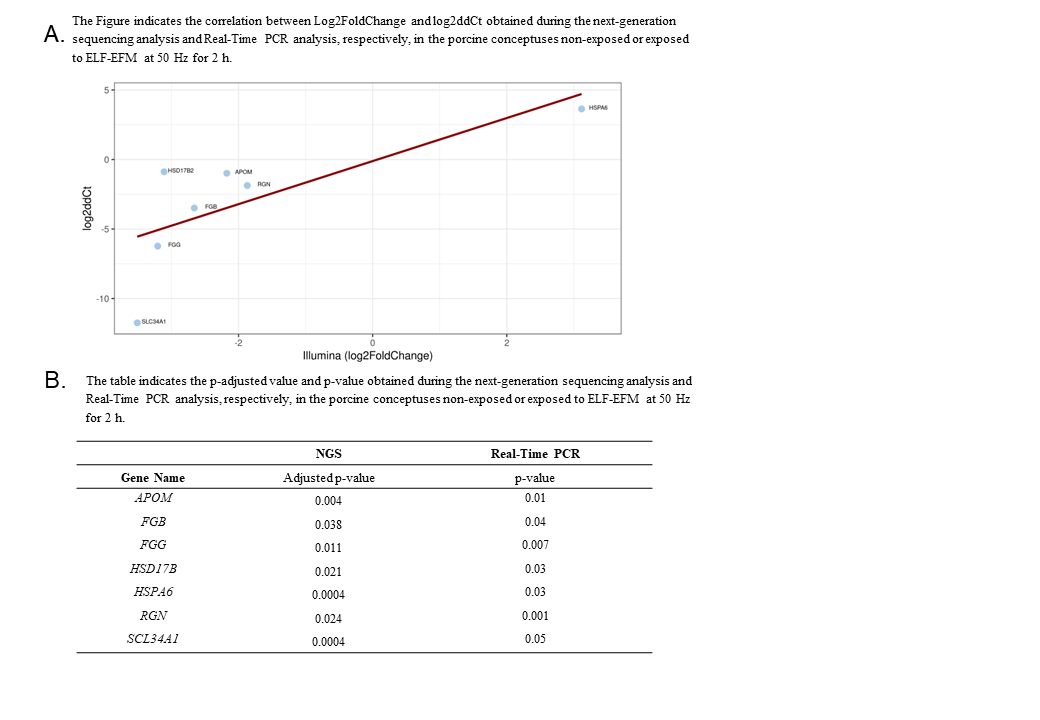

Supplement: Supplementary file 9 — Supplementary Material S8 [file 41598_2025_98918_MOESM9_ESM.png]

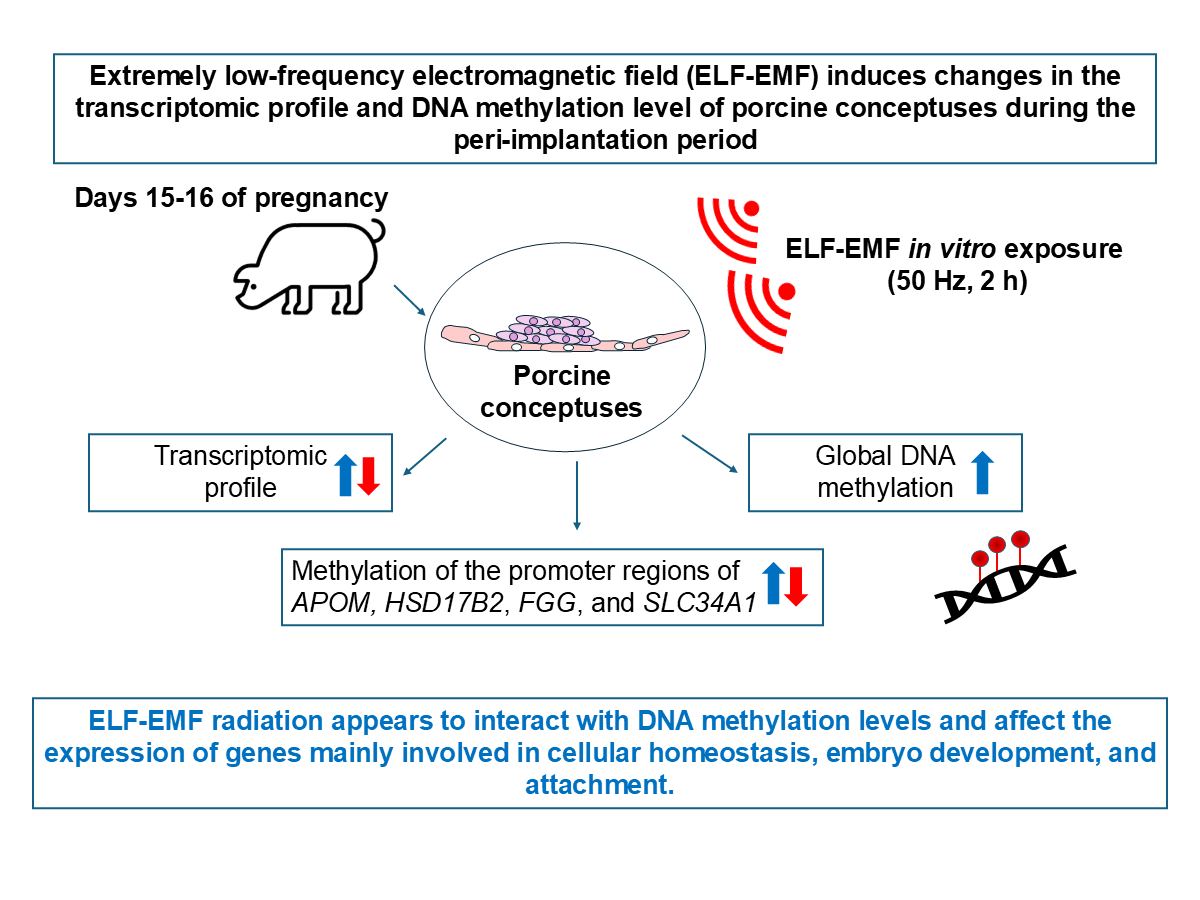

Supplement: Supplementary file 10 — Supplementary Material S9 [file 41598_2025_98918_MOESM10_ESM.tif]
