# Supplementary material for: Effect of electromagnetic field radiation on transcriptomic profile and DNA methylation level in pig conceptuses during the peri-implantation period
Source: Sci Rep. 2025 Apr 23;15:14025. doi: 10.1038/s41598-025-98918-9 (PMC12019412; doi:10.1038/s41598-025-98918-9)
Supplement: Supplementary file 11 — Supplementary Material S10 [file 41598_2025_98918_MOESM11_ESM.docx]

**Legends of supplementary information files**

**Supplementary Table S1** A full list of evaluated differentially expressed TARs in response to ELF-EMF (50 Hz, 8 mT, 2 h) in vitro treatment in the conceptuses of pigs during the peri-implantation period.

**Supplementary Table S2A** GO terms and the KEGG, REACTOME and HP pathways enriched with DEGs in ELF-EMF-treated (50 Hz, 8 mT, 2 h) conceptuses of pigs during the peri-implantation period.

**Supplementary Table S2B** The GO terms obtained during the GSEA analysis evaluated for DEGs in the ELF-EMF-treated (50 Hz, 8 mT, 2 h) conceptuses of pigs during the peri-implantation period.

**Supplementary Table S3** Predicted RNA editing sites in ELF-EMF-treated (50 Hz, 8 mT, 2 h) pig conceptuses during the peri-implantation period.

**Supplementary Table S4** GO terms and KEGG and Reactome pathways enriched with the predicted RNA editing sites in ELF-EMF-treated (50 Hz, 8 mT, 2 h) conceptuses of pigs during the peri-implantation period.

**Supplementary Table S5** Allele-specific expression candidates in ELF-EMF-treated (50 Hz, 8 mT, 2 h) conceptuses of pigs during the peri-implantation period.

**Supplementary Table S6** The relationships between genes identified in differential expression (DE), alternative splicing (AS) and single nucleotide variant (SNV) analyses, and their enriched Gene Ontology (GO) terms and KEGG pathway in ELF-EMF-treated (50 Hz, 8 mT, 2 h) conceptuses of pigs during the peri-implantation period.

**Supplementary Table S7** Supplementary Table S7 The full list of genes evaluated via Venn analysis showing ELF-EMF-induced (50 Hz, 8 mT, 2 h) changes in the transcriptomes of the conceptuses, myometrium, and endometrium of pigs during the peri-implantation period.

**Supplementary Figure 1.** Comparison of the NGS results and results obtained after validation of selected genes.

**Supplementary Figure 2.** Visualization of the summarized results obtained from ELF-EMF-treated (50 Hz, 8 mT, 2 h) pig conceptuses during the peri-implantation period.
